# Physiotherapy Management of Computer Vision Syndrome and Associated Musculoskeletal Dysfunction in Young Adults: A Systematic Review of Evidence-Based Rehabilitation Approaches

**DOI:** 10.7759/cureus.116433

**Published:** 2026-09-17

**Authors:** Mayuri R Shah, T Poovishnu Devi

**Affiliations:** 1 Neuroscience Physiotherapy, Krishna College of Physiotherapy, Krishna Vishwa Vidyapeeth (Deemed to be University), Karad, IND; 2 Cardiopulmonary Physiotherapy, Krishna College of Physiotherapy, Krishna Vishwa Vidyapeeth (Deemed to be University), Karad, IND

**Keywords:** computer vision syndrome, digital eye strain, musculoskeletal dysfunction, neck pain, physiotherapy, young adults

## Abstract

Introduction: Computer vision syndrome (CVS) is a cluster of ocular and musculoskeletal complaints linked to heavy digital device use, and a growing body of evidence ties CVS to postural strain in the neck, shoulders, and upper back. Even so, physiotherapy-based management of this combined presentation remains poorly consolidated in the literature. This review appraised the available evidence on physiotherapy interventions for CVS and its associated musculoskeletal dysfunction in young adults.

Methods: A systematic search of PubMed (National Center for Biotechnology Information, Bethesda, Maryland), PEDro (Institute for Musculoskeletal Health, University of Sydney, Sydney, Australia), Scopus (Elsevier, Amsterdam, the Netherlands), and Web of Science (Clarivate, Philadelphia, Pennsylvania) was performed for studies published between January 2020 and May 2026, supplemented by manual reference checking. Study selection followed the Preferred Reporting Items for Systematic Reviews and Meta-Analyses (PRISMA) framework through identification, screening, eligibility, and inclusion stages. Methodological quality was appraised with the PEDro scale for randomized trials, the Newcastle-Ottawa Scale for observational studies, and the Joanna Briggs Institute checklist for quasi-experimental designs. Given the anticipated heterogeneity in interventions and outcome measures, findings were synthesized narratively.

Results: Of the 1,248 records identified across the four databases, 20 studies met the eligibility criteria. Of these, 14 studies had complete and extractable data and were included in the final qualitative synthesis. Included studies evaluated cervical stabilization exercise, scapular strengthening, stretching, postural correction training, proprioceptive and motor-learning-based training, telerehabilitation, ergonomic counseling, and visual hygiene education, using tools such as the Computer Vision Syndrome Questionnaire (CVS-Q), Neck Disability Index, craniovertebral angle, Visual Analogue Scale, tear film break-up time, near point of convergence, and cervical range of motion. Physiotherapy interventions were consistently associated with reduced neck and shoulder pain, improved cervical alignment and mobility, and lower digital eye strain scores, with multimodal programs generally outperforming single-component approaches.

Conclusions: Physiotherapy-based rehabilitation appears to be a useful, non-pharmacological strategy for addressing both the visual and musculoskeletal dimensions of CVS in young adults. The current evidence base is dominated by small, short-duration studies with variable designs, underscoring the need for larger trials with standardized protocols and longer follow-up before firm clinical guidelines can be established.

## Introduction and background

Computer vision syndrome (CVS), sometimes called digital eye strain, describes a group of ocular, visual, and musculoskeletal complaints that follow extended periods spent in front of computers, smartphones, tablets, and other screen-based devices. Typical complaints include eye strain, blurred or double vision, dryness, headache, and discomfort spreading into the neck, shoulders, and upper back. These symptoms are generally attributed to a combination of sustained near-focus work, a reduced blink rate, accommodative effort, glare, poorly arranged workstations, and long periods held in a single posture. Contemporary accounts of CVS increasingly treat it as a multifactorial condition involving the ocular surface as well as the surrounding musculoskeletal system, placing it at the intersection of ophthalmology and musculoskeletal rehabilitation [1-4].

Digital dependence has grown sharply across education, work, and everyday social life, a trend accelerated by the COVID-19 pandemic, and this shift has driven a corresponding rise in CVS worldwide. A recent systematic review and meta-analysis places pooled prevalence at a substantial share of regular device users [5], making it one of the more common technology-linked health complaints of the present day. Prevalence figures are especially high among students and other young adults, particularly where screen exposure is heavy and sustained [6-8]. Increased online learning, remote work, recreational device use, and a general lack of ergonomic awareness are commonly cited drivers of this trend.

Adults aged 18-35 years represent an important population for examining CVS because of their substantial reliance on digital devices for education, employment, communication, and leisure. The consequences extend beyond visual discomfort alone, with affected individuals commonly reporting eye fatigue, headache, neck tightness, and postural fatigue; these symptoms may also interfere with concentration and academic or occupational performance [6,9,10]. However, although the present review focuses primarily on young adults aged 18-35 years, some studies addressing physiotherapy management of CVS have included broader working-age populations. Therefore, studies with wider age ranges were considered when they provided relevant evidence on physiotherapy interventions for CVS and associated musculoskeletal dysfunction, and this variation in participant age was considered when interpreting the findings.

A further strand of evidence connects CVS with musculoskeletal problems centered on the neck, shoulder girdle, and thoracic spine. Extended screen use tends to encourage sustained postures such as a forward-poking head, rounded shoulders, an exaggerated thoracic curve, and excessive neck flexion, raising mechanical demand on the cervical extensors and scapular stabilizers, altering muscle activity, straining soft tissue, and generating pain. Cross-sectional and retrospective research has repeatedly found meaningful associations between screen time or smartphone use, the severity of visual symptoms, and neck or musculoskeletal pain in student and young-adult samples [7,9,11], supporting the view of CVS as a combined visual-musculoskeletal disorder.

Historically, CVS management has centered on ophthalmological measures (artificial tears, corrective lenses, anti-glare coatings, and advice on visual hygiene), which rarely address the postural and ergonomic factors that keep symptoms recurring. Physiotherapy offers a non-pharmacological route that targets postural faults, musculoskeletal impairment, ergonomic shortcomings, and unhelpful movement habits directly. Programs built around cervical stabilization work, scapular and lower trapezius strengthening, postural retraining, and multimodal combinations of manual therapy with exercise have produced encouraging results for reducing pain, restoring cervical and shoulder alignment, and improving overall function in people who spend long hours on screens [12-14].

Despite increasing recognition of the musculoskeletal component of CVS, important gaps remain in the existing literature. The available evidence has predominantly focused on the ophthalmological and optometric aspects of CVS, including visual symptoms, ocular surface complaints, accommodative dysfunction, and visual hygiene, whereas the evidence regarding integrated physiotherapy management of the associated cervical, shoulder, and postural musculoskeletal dysfunction remains insufficiently synthesized [8,15,16]. Furthermore, existing physiotherapy studies vary considerably in the intervention components used, including therapeutic exercise, cervical stabilization, postural retraining, ergonomic education, manual therapy, and strengthening exercises. There is currently no clear consensus regarding the optimal intervention components, treatment dosage, session frequency, duration, or follow-up period, and outcome measures also vary substantially across studies. In addition, young adults aged 18-35 years remain relatively underrepresented in intervention-specific research, with several studies instead recruiting broader working-age or office-worker populations. These limitations highlight the need for a systematic synthesis specifically examining physiotherapy-based management of CVS and its associated musculoskeletal dysfunction in young adults. 

The primary objective of this systematic review was to evaluate the effectiveness of physiotherapy interventions for CVS and associated musculoskeletal dysfunction in young adults aged 18-35 years.

The secondary objectives were to:

1. Identify the physiotherapy interventions most commonly used for CVS and associated musculoskeletal dysfunction.

2. Examine the outcome measures used to assess the effects of these interventions.

3. Compare the effectiveness of multimodal physiotherapy interventions with single-component approaches.

Therefore, the aim of this systematic review was to evaluate the effectiveness of physiotherapy interventions for CVS and associated musculoskeletal dysfunction in young adults aged 18-35 years. Specifically, the review aimed to identify the physiotherapy interventions most commonly used, examine the outcome measures employed to evaluate their effects, and compare the effectiveness of multimodal and single-component approaches.

## Review

Methods

This work was conducted as a systematic review to appraise and bring together the available evidence on the effectiveness of physiotherapy interventions in managing CVS and its associated musculoskeletal dysfunction in young adults.

Search Strategy

An electronic search covering January 2020 to May 2026 was carried out across four databases: PubMed (National Center for Biotechnology Information, Bethesda, Maryland), PEDro (Institute for Musculoskeletal Health, University of Sydney, Sydney, Australia), Scopus (Elsevier, Amsterdam, the Netherlands), and Web of Science (Clarivate, Philadelphia, Pennsylvania). Search strings combined Medical Subject Headings (MeSH), free-text terms, and Boolean operators, adapted for each database's indexing conventions. Reference lists of selected articles were also hand-searched to capture additional eligible studies; this supplementary route identified a small number of frequently cited foundational studies published before 2020, which were retained given their continued relevance and citation frequency in this field. In addition to the electronic database search, the reference lists of relevant articles and included studies were manually screened to identify potentially eligible studies that may not have been retrieved through the database search.

Inclusion Criteria

Studies were included if they were peer-reviewed, full-text, and in English; evaluated physiotherapy or rehabilitation interventions relevant to CVS or associated screen-related musculoskeletal dysfunction; and assessed musculoskeletal or visual outcomes. The review primarily focused on young adults aged 18-35 years. Studies involving broader working-age populations were included only when the participant population met the predefined eligibility criteria specified in the review protocol. The age distribution of included participants is reported in Table 1 and considered in the Discussion. Randomized controlled trials, quasi-experimental studies, and observational studies relevant to physiotherapy management were all eligible for inclusion.

Exclusion Criteria

Animal studies, conference abstracts, non-English-language articles, and studies for which the full text was not accessible were excluded. Studies limited to pharmacological or ophthalmological management with no physiotherapy component, and studies conducted in pediatric or elderly populations, were also excluded. The detailed search and selection process is illustrated in Figure 1.

**Figure 1:**
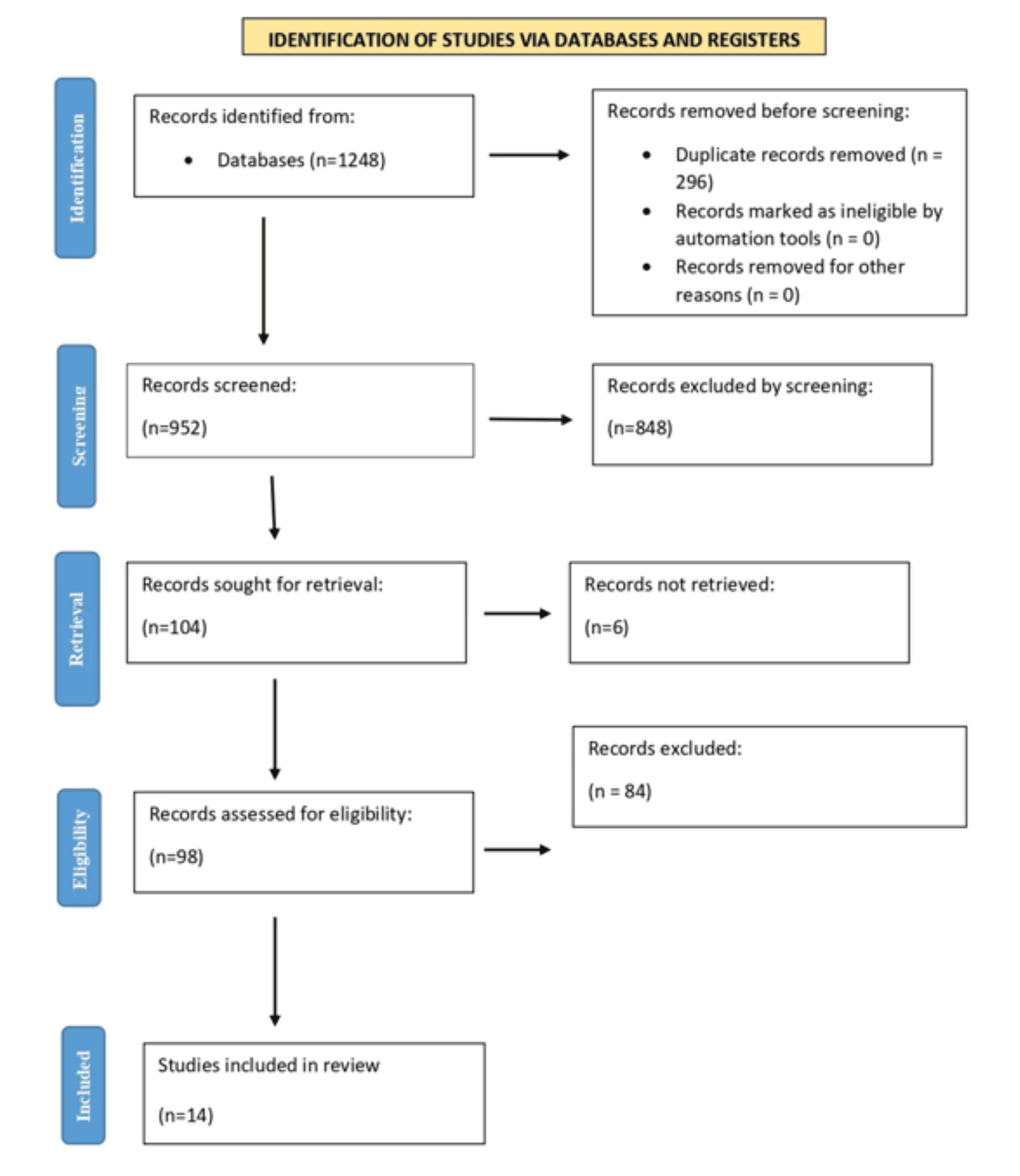
PRISMA flow diagram of the literature search results.

Data collection and analysis

Data Extraction

A single reviewer independently screened the titles and abstracts of all identified records according to the predefined inclusion and exclusion criteria, followed by full-text assessment of potentially eligible articles. The following data were extracted from each included study: study design, sample size, intervention, study duration, and outcome measures.

Assessment of Study's Risk of Bias

The methodological quality of the included studies was generally moderate to high. Among the 10 randomized controlled trials assessed using the PEDro scale, most achieved good methodological quality, although allocation concealment and blinding of therapists were the most frequently unmet criteria. One quasi-experimental study assessed using the Joanna Briggs Institute (JBI) checklist demonstrated moderate quality, while the observational and prospective studies evaluated using the Newcastle-Ottawa Scale showed satisfactory methodological quality [17,18]. Overall, the included studies were considered to have a low-to-moderate risk of bias, supporting the reliability of the evidence synthesized. However, the variability in study design and methodological quality should be considered when interpreting the findings. Table 1 shows the assessment for risk if bias. Because the included studies demonstrated substantial heterogeneity in interventions, outcome measures, and study designs, a quantitative meta-analysis and formal sensitivity analysis were not performed. The potential influence of the Editorial Expression of Concern associated with Abadiyan et al. (2021) was therefore considered qualitatively when interpreting the findings.

**Table 1:** Risk of bias assessment.

Sr. no.	Study	Assessment tool	Overall risk of bias	Main concerns
1	Rodríguez-Sanz et al. (2022)	PEDro	Low	Therapist blinding not feasible; allocation concealment unclear
2	Abadiyan et al. (2021)	PEDro	Some concerns	Editorial Expression of Concern issued; allocation concealment and blinding limitations
3	Park and Lee (2020)	PEDro	Low	Lack of therapist blinding
4	Lee et al. (2021)	PEDro	Low	Blinding of participants not possible in ergonomic intervention
5	Aneis et al. (2022)	PEDro	Low	Therapist blinding absent; relatively small sample
6	Farooq et al. (2023)	PEDro	Low	Single-blind design; therapist blinding not performed
7	Wah et al. (2021)	PEDro	Low	Minor concerns regarding allocation concealment
8	Elgendy et al. (2024)	PEDro	Low	Four-arm design; therapist blinding not feasible
9	Titcomb et al. (2023)	PEDro	Low	Participant blinding not possible
10	Argyrou et al. (2025)	PEDro	Low	No therapist blinding; short follow-up
11	Sadhwani et al. (2024)	PEDro	Low	Single-blind study; therapist blinding absent
12	Kong et al. (2017)	PEDro	Some concerns	Limited methodological reporting and allocation concealment
13	Anderson et al. (2022)	Newcastle-Ottawa Scale (NOS)	Moderate	Non-randomized prospective design; no control group
14	Nilmart et al. (2025)	PEDro	Low	Participant and therapist blinding not feasible

Results

The search of PubMed (National Center for Biotechnology Information, Bethesda, Maryland), PEDro (Institute for Musculoskeletal Health, University of Sydney, Sydney, Australia), Scopus (Elsevier, Amsterdam, the Netherlands), and Web of Science (Clarivate, Philadelphia, Pennsylvania) identified 1,248 records. After 296 duplicate records were removed, 952 records remained for title and abstract screening, of which 848 were excluded, leaving 104 records sought for retrieval. Six records could not be retrieved, and the remaining 98 reports were assessed in full text; 84 were excluded at this stage, most commonly because they addressed pharmacological or ophthalmological management without a physiotherapy component, did not report musculoskeletal outcomes, or fell outside the specified age range. Fourteen studies with complete, extractable characteristics were carried forward into the summary synthesis presented in Table 1 (Figure 1).

Demographics

The 14 studies summarized in Table 2 comprised 10 randomized controlled trials, one quasi-experimental study, one experimental study, one prospective study, one observational study, and one cross-sectional study. Sample sizes ranged from 30 to 412 participants, and intervention durations ranged from a single assessment session up to six months. These studies showed favorable effects of physiotherapy interventions on CVS and its associated musculoskeletal dysfunction. Studies revealed that cervical stabilization, scapular strengthening, postural correction, proprioceptive and motor-learning-based training, telerehabilitation exercise, and visual hygiene interventions improved neck pain, postural alignment, cervical mobility, and digital eye strain symptoms in young adults with heavy screen exposure.

Intervention Characteristics

The physiotherapy interventions varied considerably across studies and included cervical stabilization exercises, scapular strengthening, lower trapezius strengthening, ergonomic correction, manual therapy, postural correction exercises, proprioceptive training, motor learning-based rehabilitation, optimized blinking exercises, telerehabilitation, and multimodal rehabilitation programs. Intervention duration ranged from a single treatment session to six months, with most studies implementing four- to eight-week exercise programs. Outcome measures also varied substantially. The most frequently reported outcomes included neck pain intensity (Visual Analog Scale/Numeric Pain Rating Scale (VAS/NPRS)), Neck Disability Index (NDI), craniovertebral angle (CVA), cervical range of motion, CVS Questionnaire (CVS-Q), tear film break-up time, visual acuity, muscle endurance, balance, grip strength, and quality-of-life measures.

Across the included studies, physiotherapy interventions consistently demonstrated improvements in musculoskeletal symptoms, cervical posture, pain reduction, functional disability, cervical mobility, and visual symptoms. Multimodal rehabilitation programs generally produced greater improvements than single-component interventions. Table 2 contains a summary of the included studies.

**Table 2:** Summary of review of literature. CVS: Computer Vision Syndrome; NDI: Neck Disability Index; CVA: craniovertebral angle; CRA: cranial rotation angle; VAS: Visual Analog Scale; CVS-Q: Computer Vision Syndrome Questionnaire; NPRS: Numeric Pain Rating Scale.

Sr. no	Title	Aim of the study	Study design	Sample size	Study duration	Outcome measures	Intervention
1	Is cervical stabilization exercise immediately effective in patients with chronic neck pain and upper cervical spine dysfunction? (Rodríguez-Sanz et al. (2022)) [12]	To compare a single session of manual therapy plus exercise against exercise alone in the upper cervical spine (C0-C1, C1-C2, and C2-C3)	Randomized controlled trial (single-blind)	58 (29 per group)	Single session (immediate effects)	Cervical range of motion, flexion-rotation test, and pressure pain threshold	Manual therapy plus exercise vs exercise alone
2	Adding a smartphone app to global postural re-education to improve neck pain, posture, quality of life, and endurance in people with non-specific neck pain (Abadiyan et al. (2021)) [19]	To evaluate whether adding a smartphone app to global postural re-education (GPR) improves neck pain, posture, quality of life, and endurance	Randomized controlled trial (3 arms)	60 (20 per group)	Eight weeks	VAS neck pain, forward head posture/CVA, quality of life, endurance	GPR plus smartphone app vs GPR alone vs control
3	Effects of lower trapezius strengthening exercises on pain, dysfunction, posture alignment, muscle thickness and contraction rate in patients with neck pain (Park and Lee (2020)) [13]	To evaluate the added effect of lower trapezius strengthening on top of a scapular and thoracic stabilization program	Randomized controlled trial	40 (20 per group)	Four weeks (three sessions/week)	VAS, NDI, postural alignment (CVA/CRA), muscle thickness, and contraction rate	Scapular/thoracic stabilization plus lower trapezius strengthening vs stabilization alone
4	Effect of an ergonomic intervention involving workstation adjustments on musculoskeletal pain in office workers (Lee et al. (2021)) [20]	To compare pain intensity among office workers receiving an ergonomic workstation intervention versus a control group over an extended follow-up	Randomized controlled trial	Not specified in the available abstract	Assessed at 12, 24, and 36 weeks after intervention	Pain intensity (neck, shoulder, upper back, elbow, lower back, and wrist/hand)	Ergonomic workstation adjustment vs control
5	The effectiveness of a multimodal approach in the treatment of patients with upper crossed syndrome (Aneis et al. (2022)) [14]	To evaluate a multimodal approach (muscle energy technique, cervical/scapulothoracic stabilization, postural correction, and ergonomic advice) against muscle energy technique alone	Randomized controlled trial	40 (20 per group; age 30-55)	Four weeks	CVA, sagittal shoulder angle, VAS pain, and functional disability	Multimodal approach vs muscle energy technique alone
6	Effects of ELDOA and post-facilitation stretching technique on pain and functional disability in mobile users with text neck syndrome (Farooq et al. (2023)) [21]	To compare the ELDOA method with post-facilitation stretching for text neck syndrome	Randomized controlled trial (single-blinded)	40	Six weeks	NPRS, NDI, and Smartphone Addiction Scale	ELDOA technique vs post-facilitation stretching
7	Effects of proprioceptive and craniocervical flexor training on static balance in smartphone users with balance impairment (Wah et al. (2021)) [22]	To compare proprioceptive training and craniocervical flexor training for static balance and neck pain	Randomized controlled trial (double-blinded)	42	Six weeks	Balance Error Scoring System, cervical joint position sense, and VAS	Proprioceptive training vs craniocervical flexor training
8	Efficacy of head postural correction program on CVA, scapular position, and dominant hand grip strength in forward head posture subjects (Elgendy et al. (2024)) [23]	To evaluate postural correction exercise alone versus combined with scapular stabilization and/or kinesiotaping	Randomized controlled trial (4 arms)	60	Four weeks	CVA, scapular position, and hand grip strength	Postural correction exercise ± scapular stabilization ± kinesiotaping
9	The effects of postural education or corrective exercise on the CVA in young adults with forward head posture (Titcomb et al. (2023)) [24]	To compare postural education with two corrective exercise programs for forward head posture	Randomized controlled trial (4 arms)	79	Four weeks	CVA	Postural education vs self-myofascial release/stretching ± strengthening
10	The effectiveness of an exercise program based on motor learning principles for the correction of the forward head posture (Argyrou et al. (2025)) [25]	To evaluate a motor-learning-based exercise program for correcting forward head posture	Randomized controlled trial	55	Four weeks	CVA and deep neck flexor endurance	Motor-learning-based postural exercise program
11	The impact of optimized blinking on vision and related parameters in individuals with CVS (Sadhwani et al. (2024)) [26]	To evaluate optimized blinking exercises added to conventional CVS therapy	Randomized controlled trial (single-blind)	38	Six months	CVS-Q, visual acuity, near point of convergence, and tear film break-up time	Optimized blinking exercises plus conventional therapy vs conventional therapy alone
12	The effect of modified cervical exercise on smartphone users with forward head posture (Kong et al. (2017)) [27]	To evaluate whether modified cervical exercise improves cervical range of motion in smartphone users with forward head posture	Randomized controlled trial (3 arms)	Not specified in the available abstract	Four weeks	Cervical range of motion	Modified cervical exercise (three exercise variants)
13	Effects of cervical spine exercise protocol on neck pain, pericervical muscle endurance, and range of motion in medical students (Anderson et al. (2022)) [28]	To evaluate a portable device-guided cervical stretching and strengthening protocol	Prospective study	46	Six weeks	Neck pain, pericervical muscle endurance, and cervical range of motion	Device-guided cervical stretching and strengthening protocol
14	Internet-based telerehabilitation versus in-person therapeutic exercises in young adult females with chronic neck pain and forward head posture (Nilmart et al. (2025)) [29]	To compare internet-based telerehabilitation with in-person supervised therapeutic exercise	Randomized controlled trial	50	Six weeks	VAS, CVA, NDI, and cervical range of motion	Telerehabilitation exercise vs in-person supervised exercise

Discussion

The above analysis draws on studies published over the past six years on the subject of physiotherapy management for CVS and its associated musculoskeletal dysfunction in young adults. Randomized controlled trials provided the largest share of evidence, though quasi-experimental, observational, and prospective designs also contributed. There were some discrepancies among study populations, intervention protocols, and outcome measures; these are partly explained by differences in the specific screen-related condition studied (CVS, text neck syndrome, or forward head posture) and by the emphasis of each study's objectives. Nonetheless, the overall body of evidence consistently supports the value of physiotherapy-based approaches in this population.

Rodríguez-Sanz et al. [12] found that adding manual therapy to a single exercise session produced greater immediate improvement in upper cervical range of motion, the flexion-rotation test, and pressure pain threshold than exercise alone, suggesting that combining manual techniques with cervical stabilization exercise may enhance short-term outcomes in patients with upper cervical spine dysfunction. Abadiyan et al. [19] reported that adding a smartphone app to global postural re-education produced greater improvement in neck pain, posture, and endurance than GPR alone, suggesting mobile-app-based reminders and feedback may enhance adherence to postural exercise programs. (Note: the journal Trials issued an Editorial Expression of Concern for this article in January 2026; findings should be interpreted with this in mind pending its resolution.) Park and Lee [13] found that adding lower trapezius strengthening to a scapular and thoracic stabilization program produced greater improvement in neck disability and postural alignment, along with increased lower trapezius muscle thickness and contraction rate, than stabilization exercise alone. Lee et al. [20] found that an ergonomic workstation adjustment intervention reduced pain intensity in the neck, shoulder, upper back, and wrist/hand over 36 weeks of follow-up, though no reduction was seen for lower back or elbow pain, indicating that workstation modification benefits may be region-specific.

Aneis et al. [14] found that a four-week multimodal program combining muscle energy technique, cervical and scapulothoracic stabilization exercise, and postural correction training with ergonomic advice produced greater improvement in CVA, shoulder alignment, pain, and functional disability than muscle energy technique alone, supporting multimodal over single-technique approaches for upper crossed syndrome. The enrolled sample (age 30-55) was older than this review's target population, so findings should be extrapolated to younger adults with caution. Farooq et al. [21] found that both ELDOA and post-facilitation stretching reduced pain and functional disability in smartphone users with text neck syndrome, with the ELDOA method producing somewhat greater improvement, suggesting spinal-decoaptation-based stretching techniques may have a role alongside conventional stretching in device-related neck pain. Wah et al. [22] found that proprioceptive training produced greater improvement in static balance and cervical proprioception than craniocervical flexor training, while both interventions reduced neck pain, indicating that balance-oriented rehabilitation may be a useful addition to standard cervical exercise for smartphone users. Elgendy et al. [23] reported that combining postural correction exercise with scapular stabilization and kinesiotaping produced greater improvement in CVA, scapular position, and grip strength than postural correction alone, supporting multimodal exercise combinations over single-technique programs.

Titcomb et al. [24] found that corrective exercise programs produced greater improvement in CVA than postural education alone, suggesting that active exercise-based correction may outperform education-only approaches for forward head posture in young adults. Argyrou et al. [25] reported significant improvement in CVA and deep neck flexor endurance after a motor-learning-based program, with gains retained during a two-week detraining period, suggesting that incorporating attention and feedback elements into exercise may support more durable postural change. Sadhwani et al. [26] found that adding optimized blinking exercises to conventional CVS treatment produced greater improvement in visual acuity and ocular surface measures than conventional treatment alone, reinforcing the value of combining visual hygiene training with other management strategies.

Kong et al. [27] found a significant increase in cervical range of motion across all three modified-cervical-exercise groups after four weeks, indicating that even a relatively brief, low-cost exercise program can measurably improve mobility in smartphone-related forward head posture. This 2017 study predates the review's primary 2020-2026 search window and was identified through hand-searching of reference lists as a frequently cited foundational trial in this area. Anderson et al. [28] found that a six-week device-guided cervical stretching and strengthening protocol increased pericervical muscle endurance and cervical range of motion while reducing subjective neck pain in medical students, supporting the feasibility of low-cost, self-administered exercise devices for mild-to-moderate device-related neck pain. Nilmart et al. [29] found that a telerehabilitation exercise program produced improvements in pain, CVA, disability, and cervical range of motion that were comparable to an in-person supervised program, indicating that remotely delivered physiotherapy may be a viable option for young adults with chronic neck pain and forward head posture.

Collectively, these findings reinforce the interpretation that CVS extends beyond ocular discomfort to reflect a multifactorial disorder with biomechanical, neuromuscular, and behavioral components, and that physiotherapy interventions targeting postural correction, cervical stabilization, scapular strengthening, proprioceptive training, and visual hygiene can meaningfully improve both the musculoskeletal and visual dimensions of the condition. Multimodal programs that combined more than one technique generally produced larger or more consistent improvements than single-component interventions, though the limited number of large randomized trials means this observation should be treated as a pattern rather than a firm conclusion.

Age-related heterogeneity represents an important limitation of the included evidence. Although the present review primarily focused on young adults aged 18-35 years, some included studies, such as Aneis et al., recruited broader working-age populations (30-55 years). Therefore, the findings from these studies may not be fully generalizable to the predefined young-adult population. This variation in participant age should be considered when interpreting the effectiveness of multimodal physiotherapy interventions, and future studies should specifically investigate physiotherapy management of CVS in young adults aged 18-35 years. Another limitation of this review is that the study screening and selection process was conducted by a single reviewer. Although predefined eligibility criteria were applied systematically, the absence of an independent second reviewer may have increased the risk of selection bias. Future systematic reviews should involve at least two independent reviewers for title and abstract screening, full-text assessment, and data extraction to enhance methodological rigor and reduce potential bias. An Editorial Expression of Concern was issued by Trials for Abadiyan et al. in January 2026. This study was therefore interpreted cautiously in the synthesis. Although a formal sensitivity analysis was not performed because the review employed a qualitative synthesis without meta-analysis, the overall conclusions were primarily based on the broader body of evidence rather than this single study. Consequently, the potential influence of excluding Abadiyan et al. is unlikely to substantially change the overall interpretation; however, this should be confirmed by future reviews incorporating formal sensitivity analyses. Therefore, a further limitation is that a formal sensitivity analysis excluding the study by Abadiyan et al. (2021), which has an Editorial Expression of Concern, could not be performed because the review did not undertake a quantitative meta-analysis.

Clinical Implications

The findings of this review have important clinical implications for physiotherapists managing young adults with CVS. Since CVS frequently involves both visual and musculoskeletal impairments, rehabilitation should extend beyond ocular management to include posture correction, cervical stabilization, scapular strengthening, ergonomic education, and structured exercise therapy. Multimodal rehabilitation appears particularly beneficial because it simultaneously addresses biomechanical, neuromuscular, and behavioral contributors to symptoms. The evidence also supports incorporating telerehabilitation and digital exercise platforms, which may improve accessibility and treatment adherence.

Comparison with Previous Reviews

The present findings are consistent with previous literature, indicating that prolonged digital device use contributes to neck pain, forward head posture, and digital eye strain. Earlier reviews primarily emphasized prevalence and ophthalmological management, whereas this review specifically synthesizes physiotherapy-based interventions. By integrating evidence related to cervical rehabilitation, postural correction, ergonomic modification, and visual hygiene, this review provides a more comprehensive perspective on rehabilitation strategies for CVS.

Future Research

Future studies should focus on adequately powered multicenter randomized controlled trials using standardized intervention protocols and validated outcome measures. Long-term follow-up is required to determine whether improvements in posture and digital eye strain are sustained. Future research should also investigate optimal exercise dosage, adherence strategies, and the effectiveness of combining physiotherapy with emerging digital health technologies.

## Conclusions

This systematic review highlights CVS as a significant digital-health concern among young adults, driven by the widespread and prolonged use of digital devices for academic, occupational, and recreational purposes. The evidence reviewed here demonstrates a strong association between CVS and musculoskeletal dysfunction, particularly in the cervical spine, shoulder girdle, and upper thoracic region. The findings indicate that physiotherapy interventions, including cervical stabilization exercise, scapular strengthening, postural correction training, proprioceptive and motor-learning-based training, telerehabilitation, ergonomic education, and visual hygiene strategies, are effective in reducing digital eye strain symptoms, improving postural alignment, decreasing neck pain, and enhancing functional outcomes. The available evidence supports physiotherapy as an essential component of multidisciplinary CVS management. However, the absence of standardized intervention protocols and the limited number of large, high-quality randomized controlled trials highlight the need for further rigorous research to establish evidence-based clinical guidelines.
